# The ALS-linked E102Q mutation in Sigma receptor-1 leads to ER stress-mediated defects in protein homeostasis and dysregulation of RNA-binding proteins

**DOI:** 10.1038/cdd.2017.88

**Published:** 2017-06-16

**Authors:** Alice Dreser, Jan Tilmann Vollrath, Antonio Sechi, Sonja Johann, Andreas Roos, Alfred Yamoah, Istvan Katona, Saeed Bohlega, Dominik Wiemuth, Yuemin Tian, Axel Schmidt, Jörg Vervoorts, Marc Dohmen, Cordian Beyer, Jasper Anink, Eleonora Aronica, Dirk Troost, Joachim Weis, Anand Goswami

**Affiliations:** 1 Institute of Neuropathology, RWTH Aachen University Medical School, Aachen, Germany; 2 Institute of Biomedical Engineering, Deparment of Cell Biology, RWTH Aachen University Medical School, Aachen, Germany; 3 Institute of Neuroanatomy, RWTH Aachen University Medical School, Aachen, Germany; 4 Leibniz-Institut für Analytische Wissenschaften – ISAS – e.V., Dortmund, Germany; 5 Institute of Genetic Medicine, John Walton Muscular Dystrophy Research Centre, International Centre for Life, Central Parkway, Newcastle upon Tyne, England, UK; 6 Department of Genetics, King Faisal Specialist Hospital and Research Centre, Riyadh, Saudi Arabia; 7 Institute of Physiology, RWTH Aachen University Medical School, Aachen Germany; 8 Institute of Biochemistry and Molecular Biology, RWTH Aachen University Medical School, Aachen, Germany; 9 Department of (Neuro)Pathology, Academic Medical Center, University of Amsterdam, Amsterdam, The Netherlands

## Abstract

Amyotrophic lateral sclerosis (ALS) is characterized by the selective degeneration of motor neurons (MNs) and their target muscles. Misfolded proteins which often form intracellular aggregates are a pathological hallmark of ALS. Disruption of the functional interplay between protein degradation (ubiquitin proteasome system and autophagy) and RNA-binding protein homeostasis has recently been suggested as an integrated model that merges several ALS-associated proteins into a common pathophysiological pathway. The E102Q mutation in one such candidate gene, the endoplasmic reticulum (ER) chaperone Sigma receptor-1 (SigR1), has been reported to cause juvenile ALS. Although loss of SigR1 protein contributes to neurodegeneration in several ways, the molecular mechanisms underlying E102Q-SigR1-mediated neurodegeneration are still unclear. In the present study, we showed that the E102Q-SigR1 protein rapidly aggregates and accumulates in the ER and associated compartments in transfected cells, leading to structural alterations of the ER, nuclear envelope and mitochondria and to subsequent defects in proteasomal degradation and calcium homeostasis. ER defects and proteotoxic stress generated by E102Q-SigR1 aggregates further induce autophagy impairment, accumulation of stress granules and cytoplasmic aggregation of the ALS-linked RNA-binding proteins (RBPs) matrin-3, FUS, and TDP-43. Similar ultrastructural abnormalities as well as altered protein degradation and misregulated RBP homeostasis were observed in primary lymphoblastoid cells (PLCs) derived from E102Q-SigR1 fALS patients. Consistent with these findings, lumbar *α*-MNs of both sALS as well as fALS patients showed cytoplasmic matrin-3 aggregates which were not co-localized with pTDP-43 aggregates. Taken together, our results support the notion that E102Q-SigR1-mediated ALS pathogenesis comprises a synergistic mechanism of both toxic gain and loss of function involving a vicious circle of altered ER function, impaired protein homeostasis and defective RBPs.

Several familial ALS (fALS) and frontotemporal dementia (FTD)-linked mutations affect protein homeostasis and autophagy, including ubiquilin-2 (*UBQLN2*), *p62/SQSTM1* (sequestosome1), optineurin (*OPTN*), TANK-binding kinase 1 (*TBK1*), valosin-containing protein (*VCP*), charged multivesicular body protein 2B (*CHMP2B*), vesicle-associated membrane protein B (*VAPB*) and *FIG4*.^1, 2^ Recent studies emphasized a functional interplay between autophagy and RNA processing, associating several ALS-FTD genes in a converging pathway leading to insufficient degradation and abnormal aggregation of proteins.^2^ Specifically, mutations in RBPs such as Tar DNA-binding protein-43 (TDP-43), fused in sarcoma (FUS), survival of motor neuron-1 (SMN1), ataxin-2 (ATX2), optineurin (OPT), angiogenin (ANG) and matrin-3 (MATR3) cause fALS with distinct aggregates,^3^ disturbed autophagy and altered RNA processing. Even in sporadic ALS (sALS), toxic aggregates of such proteins in MNs are frequent and can be triggered by defective UPS/autophagy.^4^

Aggregation of these RBPs together with other proteins is physiological and reversible (unfolded protein response, UPR).^5^ RBPs control RNA polymerase elongation, mRNA maturation, transport and degradation, and regulate transcriptional activity and distribution of RNAs by RNA granule formation. Among the latter, stress granules (SGs) are generated in response to stressful conditions. Prolonged SG formation affects protein quality control and vital cellular processes like apoptosis, signalling and RNA decay.^5^

Sigma receptor-1 (SigR1) is an ER chaperone involved in neuronal survival, ion channel activity, Ca^2+^ signalling, synaptic plasticity, memory and drug addiction.^6^ The E102Q-SigR1 missense mutation causes fALS;^7^ a splice site mutation (c.151+1G>T)^8^ and recently the homozygous E138Q and E150K mutations^9^ have been linked to autosomal recessive distal hereditary motor neuropathy (dHMN). Moreover, SigR1 is involved in several neurodegenerative disorders including Parkinson's, Alzheimer's and Huntington's disease.^10^

We previously described abnormal modification as well as altered localization of SigR1 protein in sALS patient spinal cord.^11^ Mavlyutov *et al.* demonstrated that lack of SigR1 exacerbates ALS progression in G93A-SOD1 mice.^12^ SigR1^−^^/−^ mice showed MND pathology and symptoms.^13^ *In vitro*, SigR1 depletion causes Ca^2+^ dysregulation, autophagy defects and ER stress-mediated neuronal death.^11, 14^ Furthermore, a SigR1 agonist improved motor function and MN survival in SOD1 mice.^15^ These studies suggest a crucial role of SigR1 for neuronal survival. However, it is still unclear whether E102Q-SigR1 causes ALS due to a gain or loss of functions.

Using PLCs obtained from E102Q-SigR1 fALS patients^7^ and autopsy specimens from sALS and fALS patients as well as cell culture models, we found that E102Q-SigR1 aggregates induce ER stress with distinct ER and nuclear envelope alterations, impaired Ca^2+^ homeostasis and autophagy pathways and aberrant extra-nuclear localization of several ALS-associated RBPs. Our results support the notion that mutant (m) SigR1 induces neuronal toxicity by a loss of normal function combined with a toxic gain of abnormal functions.

## Results

### mSigR1 aggregation and cellular toxicity

**Figure 1:**
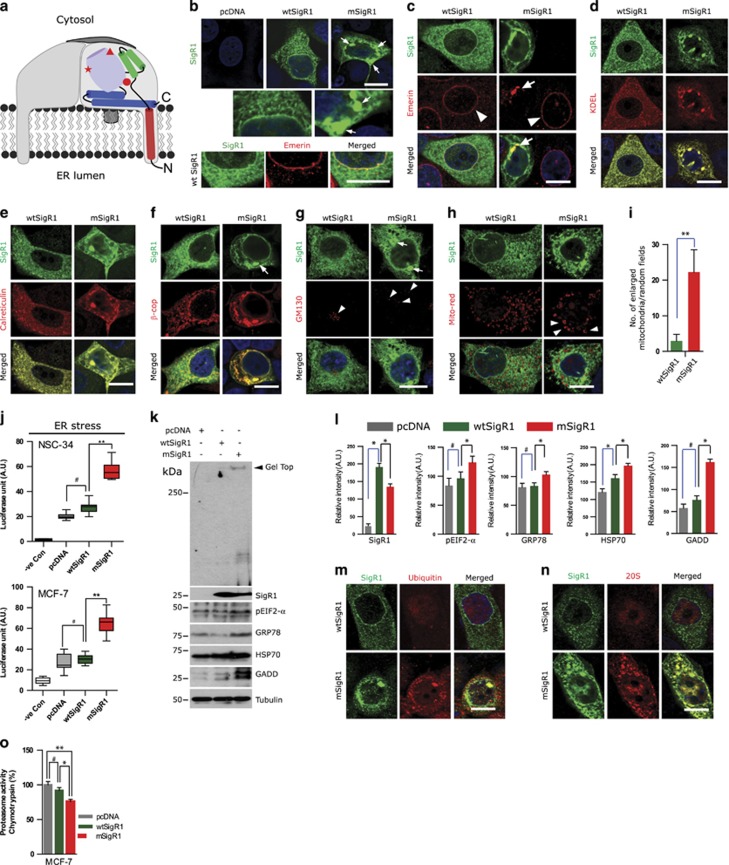
mSigR1 abnormally accumulates in the ER and induces cellular toxicity. (**a**) Model of the homotrimeric SigR1 (based on ref. 16). Subunits are represented in gray, secondary structure of one subunit is color-coded; each monomer is composed of an amino-terminal transmembrane domain (red) which crosses the ER membrane from lumen to cytosol. The transmembrane domain is followed by two *α*-helices (green) that lead to a *β*-barrel (light violet), the putative ligand binding domain. Two carboxy-terminal *α*-helices (blue) form a hydrophobic membrane-embedded surface. The location of the critical residue Glu-102 (E102Q) is marked by a red dot. Red star and red triangle represent two recently discovered mutations.^9^ (**b**) Reticular pattern of SigR1 staining combined with a distinct nuclear envelope localization (lower panel) in wtSigR1-transfected MCF-7 cells; globular mSigR1 aggregates (arrows; see also enlargement, middle row) in MCF-7 cells expressing mSigR1. Immunofluorescence; scale bars, 15 *μ*m. (**c**) Co-immunofluorescence of wtSigR1 and mSigR1 with emerin as a nuclear envelope marker in MCF-7 cells (arrowheads). Note that the focal emerin accumulations (arrows) co-localize with SigR1 aggregates. Scale bar, 10 *μ*m. (**d** and **e**) Co-localization of wtSigR1 and mSigR1 with the ER markers KDEL and (**e**) calreticulin in MCF-7 cells. Scale bar, 15 *μ*m. (**f** and **g**) Co-labelling of SigR1 with the *β*-cop (ER-Golgi-associated compartments) and GM130 (Golgi marker). Note the co-localization of mSigR1 with *β*-cop and the Golgi dispersal (arrowheads) in mSigR1 expressing MCF-7 cells. Scale bar, 10 *μ*m. (**h**) Co-immunofluorescence of wtSigR1 and mSigR1 with mito-red as a marker for mitochondria in MCF-7 cells. Scale bar, 10 *μ*m. (**i**) Quantification of the mito-red staining depicted in (**h)** showing numbers of enlarged mitochondria/random field of view. (**j**) NSC34 and MCF-7 cells were co-transfected either with empty pcDNA vector, wtSigR1 or mSigR1 together with the luciferase gene downstream of the ERSE promoter. After 48 h, luciferase units (AU) were analyzed as a measure of ER stress. *P<0.05,**P<0.005, #not significant. (**k**) MCF-7 cells were transfected with pcDNA, wtSigR1 or mSigR1 as described above and analyzed for ER stress and UPR induction by immunoblotting. (**l**) Quantification of the band intensities normalized with *α*-tubulin depicted in **k**. Values represent the mean±S.D. of three independent experiments. *P<0.05*.* (**m**) Ubiquitin immunoreactivity of wtSigR1 and mSigR1 in MCF-7 cells. Scale bar, 10 *μ*m. (**n**) Co-localization of mSigR1 aggregates with the accumulated 20s subunit of the proteasome in MCF-7 cells expressing mSigR1. Scale bar, 10 *μ*m. (**o**) Chymotrypsin-like proteasomal activity assays were performed using MCF-7 cells transfected with pcDNA, wtSigR1 and mSigR1, as described in material and methods. Note the significant decrease in proteasome activity due to mSigR1 expression when compared to wtSigR1 and pcDNA. Means±S.D. of three independent experiments each performed in triplicate. *P<0.05, **P<0.005*,* # not significant

MCF-7 cells normally express little or no SigR1 (Supplementary Figure 1A). Over-expressed wtSigR1 showed localization in the ER (Figures 1d and e), nuclear envelope (Figures 1b and c) and ER-Golgi intermediate compartment (Figure 1f) of transiently transfected MCF-7 cells. mSigR1 formed ER-associated aggregates (Figures 1b and d-f and Supplementary Figures 1B and C) combined with disintegrated ER membranes and overall cell morphology (Figure 1b,Supplementary Figure 1B). Shuttling of the nuclear envelope protein Emerin was impaired (Figure 1c). These results comply with the recently published crystal structure of SigR1, suggesting that E102Q mutation favors SigR1 aggregation and toxicity.^16^ mSigR1 aggregates did not associate with the Golgi marker GM130, but there was Golgi dispersal (Figure 1g). SigR1 is abundant at the ER-mitochondria-associated membrane (MAM).^6^ Immunoreactivity for the mitochondrial marker Tim23 was reduced, even though few enlarged mitochondria (or clusters of mitochondria) co-localized with SigR1 aggregates (Supplementary Figure 1H). However, consistent with a recent report,^9^ mitochondria overall did not associate significantly with these aggregates, confirmed by mito-red staining (Figures 1h and i).

ERSE reporter assay showed increased ER stress in both NSC-34 and MCF-7 cells (Figure 1j) expressing mSigR1. Immunoblotting revealed gel top smear (Figure 1k) and significantly increased levels of the ER stress markers GRP78, pEIF2-*α*, GADD and HSP70 (Figures 1k and l) which correlated well with the expression level of mSigR1 in a dose-dependent manner (Supplementary Figures 1D–G). RT-PCR showed a mild, but statistically insignificant increase in RNA of the ER stress markers ATF4 and ATF6 (Supplementary Figures 2A–C), whereas E102Q-SigR1 PCLs showed a significant increase in ATF4, but no ATF6 RNA expression (Supplementary Figure 2D; see below).

Co-localization of Ubiquitin-positive mSigR1 aggregates (Figure 1m) with 20S proteasome subunits (Figure 1n) suggest that they were targeted for UPS-mediated degradation and interfered with the UPS machinery. Accordingly, chymotrypsin-like proteasome activity (Figure 1o and Supplementary Figure 1I) was significantly reduced compared to cells expressing empty vector (pcDNA) or wtSigR1.

### E102Q-SigR1 fALS patients

**Figure 2:**
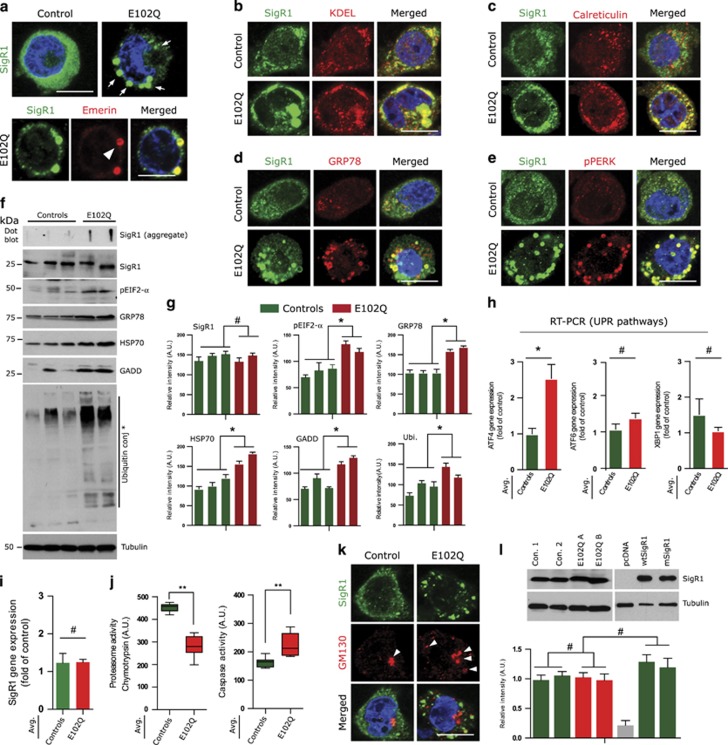
mSigR1 is abnormally accumulated in the ER and induces cellular toxicity in E102Q-SigR1 fALS patient lymphoblastoid cells. (**a**) Immunoreactivity of globular SigR1 aggregates (arrows) in E102Q-SigR1 fALS patient lymphoblastoid cells compared to the healthy control. Note the co-localization of SigR1 aggregates with the nuclear envelope marker emerin (arrowhead). Scale bar, 15 *μ*m. (**b** and **c**) Co-localization of mSigR1 aggregates with the ER markers KDEL and (**c**) calreticulin in E102Q-SigR1 fALS patient lymphoblastoid cells compared to the healthy control. Scale bars, 15 *μ*m. **(d** and **e)** Upregulation and co-localization of the ER stress markers GRP78 (**d**) and pPERK (**e**) with mSigR1 aggregates in E102Q-SigR1 fALS patient lymphoblastoid cells compared to the healthy control. Scale bars, 15 *μ*m. (**f**) Immunoblot analysis of SigR1 and established ER stress and UPR markers in healthy control and E102Q-SigR1 fALS lymphoblastoid cell lysates. Dot blot analysis (top) shows the presence of triton-X insoluble aggregates in E102Q-SigR1 fALS lymphoblastoid cells. (**g**) Quantification of the band intensities normalized with *α*-tubulin depicted in **f**. Values represent the mean±S.D. of three independent experiments. *P<0.05*.* (**h–i**) RT-PCR analysis of the UPR pathways in three healthy control lymphoblastoid cell lines compared to two E102Q-SigR1 fALS patient lymphoblastoid cell lines. E102Q-SigR1 fALS patient’s lymphoblastoid cells showed a significant increase in ATF4 mRNA expression. *P<0.05, # not significant. Note that SigR1 gene expression does not differ between controls and E102Q-SigR1 fALS patients. (**j**) E102Q-SigR1 fALS patient’s lymphoblastoid cell (*n*=2) and control lymphoblastoid cell (*n*=3) lysates were subjected to chymotrypsin-like proteasomal activity and caspase-3 activity assays as described in material and methods. Values are derived from the average of three control lymphoblastoid cell lines compared to average of two E102Q-SigR1 fALS patient’s lymphoblastoid cell lines from three independent experiment,**P<0.005*.* (**k**) GM130 and SigR1 immunolabelling in E102Q-SigR1 fALS and control lymphoblastoid cells. Scale bar, 15 *μ*m. (**l**) Immunoblot analysis using SigR1 antibody to compare SigR1 levels in lymphoblastoid cells from E102Q-SigR1 patient lymphoblasts and healthy controls as well as transiently transfected MCF-7 cells. Quantification of the band intensities normalized with *α*-tubulin is shown below. (#) denotes absence of significant differences

We generated immortalized PLCs from blood samples of E102Q-SigR1 fALS patients.^7^ Interestingly, these cells also showed ER-associated mSigR1 aggregates (Figures 2a–c) which co-localized with accumulated Emerin (Figure 2a). mSigR1 aggregates also co-localized with several ER stress markers (Figures 2d and e). Dot blot and immunoblot analysis revealed triton-X insoluble gel top aggregates and increased levels of GRP78 and pEIF2-*α* (Figures 2f and g). Elevated levels of ubiquitin conjugates, HSP70 and GADD further indicated proteotoxic stress (Figures 2f and g). Accordingly, both PLCs showed significantly elevated ATF4 mRNA expression (Figure 2h and Supplementary Figure 2D). mRNAs of other UPR branches (ATF6, XBP1) remained unchanged (Figure 2h and Supplementary Figure 2D). Most importantly, SigR1 mRNA expression showed no significant difference between E102Q-SigR1 and control PLCs (Figure 2i).

**Figure 3:**
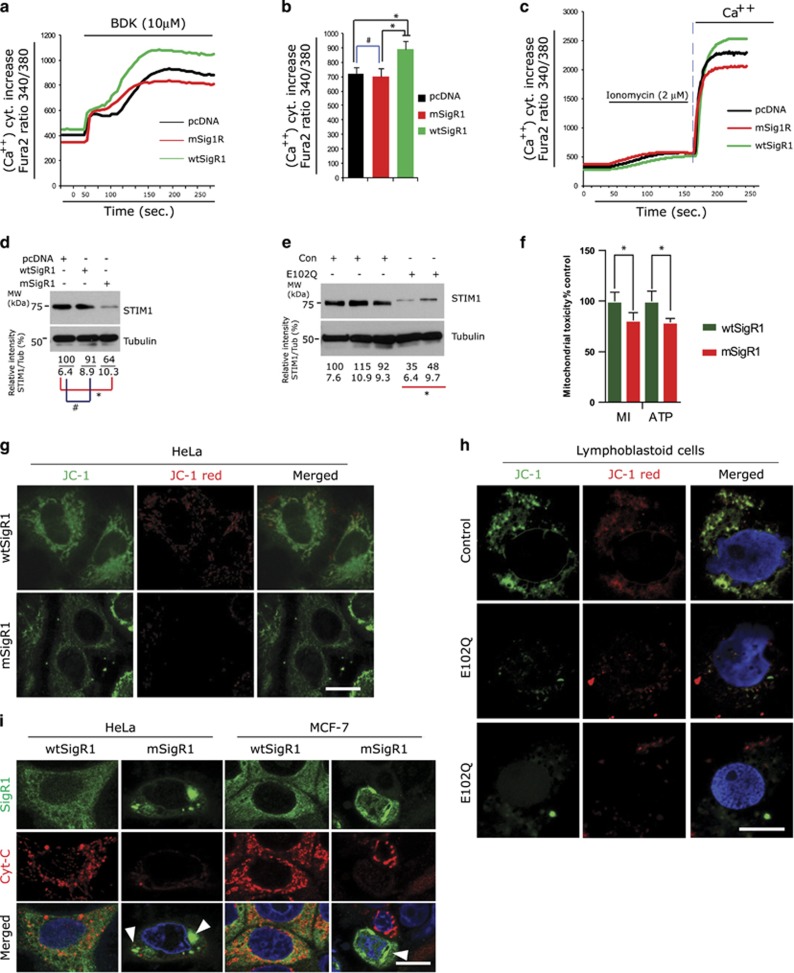
mSigR1 induces mitochondrial toxicity and fails to mobilize IP3R and SOCE-mediated Ca^2+^ signalling. (**a** and **b**) MCF-7 cells were transiently transfected as previously described; 48 h later cells were loaded with Fura-2AM for 30 min, washed twice and then stimulated with 10 *μ*M BDK under Ca^2+^-free conditions. Average traces of the BDK-induced increase in [Ca^2+^]^i^ from the ER store through IP3R are represented. Note the increase of [Ca^2+^]^i^ in wtSigR1-transfected cells (green curve) compared to a significant decrease in mSigR1-transfected cells (red curve). Results are expressed as mean±S.E.M. of ~30 cells. Mean changes in peak [Ca^2+^]^i^ measured are given. The asterisks denote a statistically significant difference (*P<0.05). (**c**) Representative SOCE elicited in transfected cells loaded with Fura-2AM as described above. SOCE was triggered by the addition of 2 *μ*M ionomycin under Ca^2+^-free conditions. SOCE developed (vertical dot line) after the addition of Ca^2+^ is depicted. (**d**) STIM1 immunoblot analysis from lysates obtained from pcDNA, wtSigR1 and mSigR1-transfected MCF-7 cells. Note the significant down-regulation of STIM1 in mSigR1-transfected cells. The fold change below represents the quantification of band intensities normalized against *α*-tubulin. Values derived from three independent experiments. *P<0.05*.* (**e**) Significantly decreased STIM1 levels in E102Q-SigR1 fALS lymphoblastoid cell lysates compared to healthy control lymphoblastoid cells. The fold change below represents the quantification of band intensities normalized against *α*-tubulin. Values derived from three independent experiments. *P<0.05*.* (**f**) Significantly reduced mitochondrial membrane integrity and ATP production in mSigR1 expressing MCF-7 cells compared to wtSigR1 expressing cells measured by the tox glow assay. Values derived from three independent experiments. *P<0.05*.* (**g**) JC-1 staining of HeLa cells transfected with wtSigR1 or mSigR1. Note the reduced mitochondrial potential in mSigR1 expressing cells. Scale bar, 10 *μ*m. (**h**) JC-1 staining of lymphoblastoid cells obtained from E102Q-SigR1 fALS patients and healthy controls. Note the decreased membrane potential in mSigR1 expressing cells. Scale bar, 10 *μ*m. (**i**) Cytochrome C immunolabelling of HeLa and MCF-7 expressing wtSigR1 or mSigR1. Note the Cytochrome C release in cells showing mSigR1 aggregates (arrowheads). Scale bar, 10 *μ*m

Decreased proteasomal and increased caspase-3 activity was consistent with these observations (Figure 2j and Supplementary Figure 2E). GM130 staining revealed significant Golgi dispersal compared to the controls (Figure 2k). mSigR1 aggregates did not co-localize significantly with mitochondria (not shown); however, mitochondrial labelling (JC-1 dye) was significantly reduced (see below, Figure 3h). Importantly, SigR1 protein levels actually did not differ significantly between the E102Q-SigR1 PLCs and our transiently transfected cell lines (Figure 2l); the results obtained from the E102Q-SigR1 PLCs and the transiently transfected cell lines were consistent throughout, indicating that the latter were not due to over-expression artefacts.

### mSigR1 impairs overall Ca^2+^ homeostasis and induces mitochondrial toxicity

SigR1 is abundant at the MAM and regulates ER-mitochondria Ca^2+^ transmission.^6^ mSigR1 induces mitochondrial toxicity^17^ and disturbs ER-mitochondria calcium exchange.^9^ During ER stress and ER Ca^2+^-deprivation, SigR1 translocates to the plasma membrane to regulate store-operated Ca^2+^ entry (SOCE) together with channel proteins like STIM1 and Orai1.^18^ Expression of wtSigR1 in our MCF-7 cells efficiently induced IP3R-mediated Ca^2+^ release from the ER (Figures 3a and b). mSigR1, however, failed to trigger efficient Ca^2+^ release (Figures 3a and b), confirming a recent report.^9^ Ionomycin treatment to activate SOCE (Figure 3c) led to significantly increased intracellular Ca^2+^ in cells transfected with wtSigR1, but not with mSigR1 (Figure 3c). Accordingly, STIM1 protein levels were significantly reduced in MCF-7 cells expressing mSigR1 and in E102Q-SigR1 PLCs (Figures 3d and e).

Ca^2+^ homeostasis and mitochondrial functions are closely linked. Using the sensitive Tox glow assay we observed significantly impaired mitochondrial membrane integrity (MI) and decreased ATP production in mSigR1-transfected cells (Figure 3f). Consistent with the reduced number of (albeit enlarged) mitochondria (Figure 1h) and reduced Tim23 immunoreactivity in mSigR1-expressing cells (Supplementary Figure 1H), the mitochondrial membrane potential was significantly decreased (disappearance of the red-dotted color in the JC-1 stain) in both mSigR1-expressing cells (Figure 3g) and E102Q-SigR1 PLCs (Figure 3h). This led to Cyt-C release (reduced Cyt-C staining, Figure 3i; arrowheads) in mSigR1-transfected HeLa and MCF-7 cells.

### Structural ER and mitochondria alterations and accumulation of autophagic material

**Figure 4:**
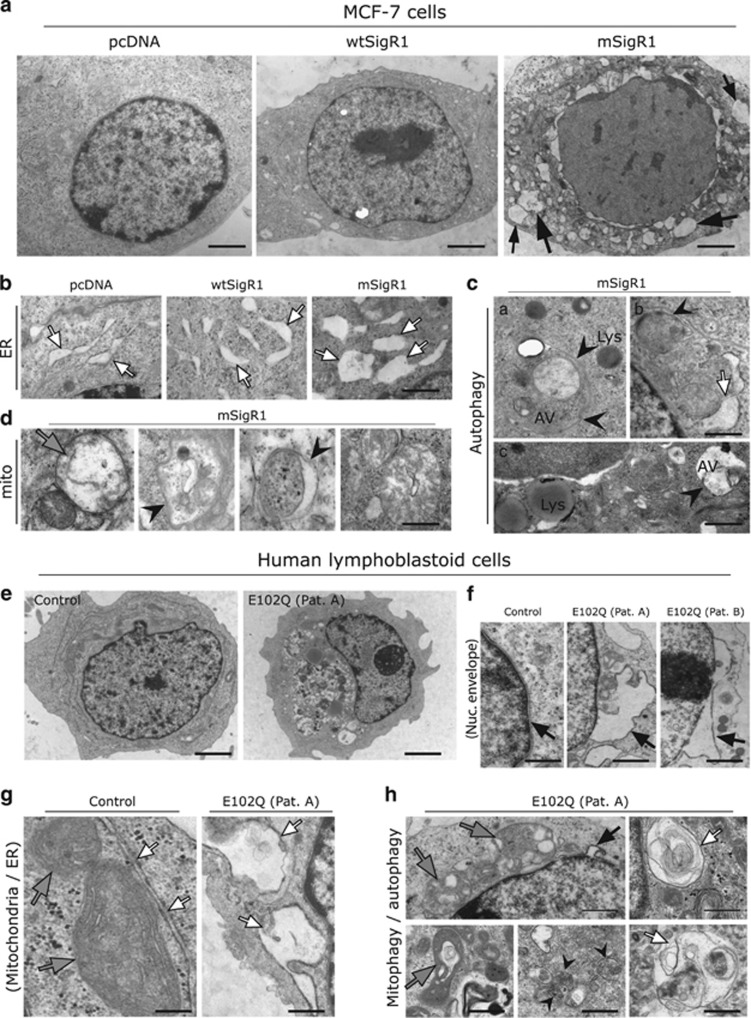
mSigR1 leads to structural abnormalities of ER and mitochondria. (**a**) MCF7 cells expressing pcDNA, wtSigR1 or mSigR1 were fixed with 2.5% buffered glutaraldehyde and processed for EM. Several membrane-bound vacuolar structures (black arrows) probably derived from the ER in a representative mSigR1-expressing cell compared to cells expressing wtSigR1 and pcDNA control. Scale bars, 1 *μ*m. (**b**) Higher magnification showing the widened ER (arrows in right panel) in representative mSigR1-expressing cells compared to pcDNA and wtSigR1-transfected cells. Scale bar, 0.5 *μ*m. (**c**) Higher magnification showing double membrane autophagic vacuoles in representative mSigR1-expressing MCF-7 cells. (a) Large autophagosome (AV; arrowheads) containing mitochondria and other structures in close proximity to lysosomes (Lys); (b) autophagosome (arrowhead) and widened ER (white arrow); (c) autophagosome (AV) and lysosomes (Lys). Scale bar, 0.5 *μ*m. (**d**) Enlarged mitochondria (arrows) showing abnormal cristae architecture, some undergoing mitophagy (arrowheads) in mSigR1-transfected cells. Scale bar, 0.4 *μ*m. (**e**) Primary lymphoblastoid cells from healthy control and E102Q-SigR1 fALS patients were fixed with 2.5% buffered glutaraldehyde and processed for EM. Control lymphoblasts show an overall normal ultrastructure, whereas E102Q-SigR1 fALS patient lymphoblastoid cells reveal prominent accumulation of autophagic material. Scale bars, 2.5 *μ*m. (**f**) Prominent nuclear envelope (arrows) protrusions in E102Q-SigR1 fALS lymphoblastoid cells and rather normal nuclear envelope in control lymphoblastoid cells. Scale bar, 0.5 *μ*m. **(g)** Normal ER (white arrows) and mitochondria (gray arrows) in healthy control lymphoblastoid cells; E102Q-SigR1 fALS patient lymphoblastoid cells showing overall widened ER (arrows). Scale bar, 0.5 *μ*m. (**h**) E102Q-SigR1 fALS lymphoblasts displaying vacuolar degeneration of mitochondria (gray arrows), multivesicular bodies (arrowheads) and autophagosomes filled with membranous and granular material (white arrows). Black arrow: nuclear envelope protrusion. Scale bars, 0.4 *μ*m

EM revealed numerous vacuoles in mSigR1-expressing cells (Figure 4a and Supplementary Figure 3A) many of which were probably ER-derived and might actually be precursors of autophagic vacuoles,^19^ often containing non-degraded autophagic substrates (Figure 4a and Supplementary Figure 3A). There was prominent ER swelling (Figure 4b, arrows) compared to the pcDNA control and wtSigR1 over-expressing cells. Higher magnification revealed several autophagic vacuoles with characteristic double membranes and sequestered cellular components in mSigR1-transfected cells (Figure 4c and Supplementary Figure 3B; arrowheads). Furthermore, expression of mSigR1 induced ER widening at the MAM (Supplementary Figure 3C and Figure 4c, arrow) and led to abnormalities of mitochondrial morphology (swelling of mitochondria, mitophagy) (Figure 4d, arrowheads). In contrast, wtSigR1 over-expressing cells showed rather normal ER, mitochondria and MAM (Figure 4b and Supplementary Figure 3C; arrows). Consistent with the nuclear envelope defects seen previously, expression of mSigR1 induced prominent outfoldings of the nuclear envelope (Supplementary Figure 3D). EM of E102Q-SigR1 PLCs revealed similar ultrastructural defects (Figures 4e–h); control PLCs showed rather normal ER, mitochondria and MAM (Figure 4g).

### mSigR1 induces defective autophagy

Autophagy largely relies on intact ER structure and functions. We showed morphological ER alterations, disturbed intracellular Ca^2+^ homeostasis and accumulations of autophagic vacuoles in both mSigR1-expressing cells and in E102Q-SigR1 PLCs; thus we hypothesized that mSigR1 might impair autophagic processes. Autophagy can be monitored at different stages by several well-established methods.^14, 20, 21^

**Figure 5:**
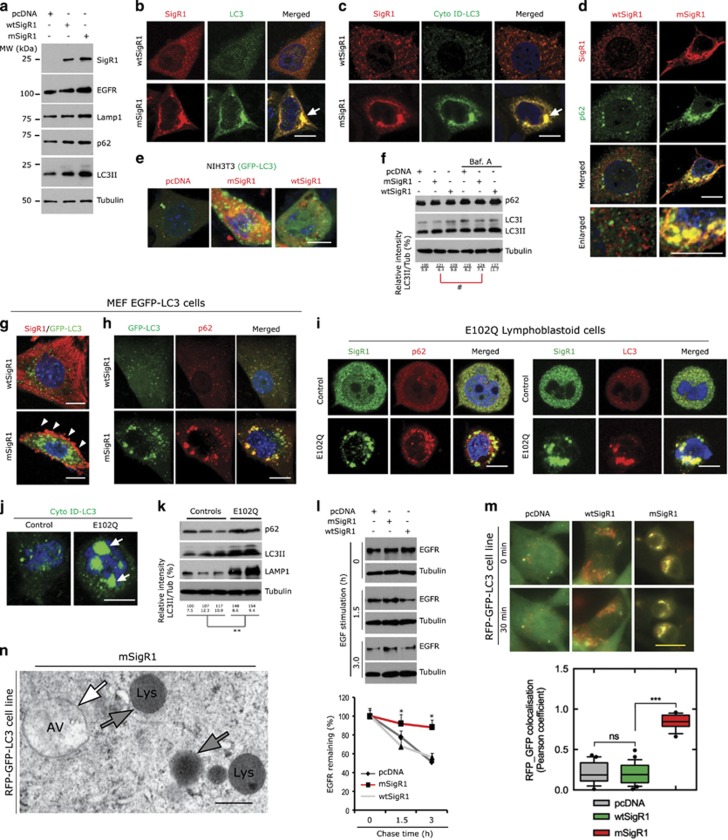
mSigR1 leads to defective autophagy and autophagosome-lysosome fusion. (**a**) Immunoblot analysis for autophagy markers in A431 cells transiently transfected with pcDNA, wtSigR1 and mSigR1. Note the increased levels of autophagy markers in mSigR1 expressing cells. (**b**–**d**) MCF-7 cells were transiently transfected with wtSigR1 or mSigR1 and then processed for (**b**) co-immunolabelling using SigR1 and LC3 antibodies, (**c**) co-immunolabelling using SigR1 and Cyto-ID dye (for labelling autophagosomes) and (**d**) co-immunolabelling using SigR1 and p62 antibodies. Scale, 10 *μ*m. (**e**) Increased accumulation of autophagosomes in the stable autophagy reporter cell line NIH3T3-GFP transfected with mSigR1. Green: GFP-LC3; red: SigR1; scale bar, 10 *μ*m. (**f**) Immunoblot analysis of NIH3T3-GFP-LC3 cells transfected with wtSigR1 or mSigR1 and additionally treated with the autophagy inhibitor Bafilomycin A for 2 h. Note the unchanged LC3-II levels in mSigR1-transfected cells after Bafilomycin A treatment. Corresponding densitometric data are shown at the bottom; where the upper number represents the relative LC3II/Tub levels and the lower numbers are the S.D. The asterisks (*) denote significant differences (*P<0.05), while # denotes absence of a significant difference. (**g** and **h**) Primary fibroblasts isolated from autophagy reporter GFP-LC3 transgenic mice were transfected with wtSigR1 or mSigR1 and immuno-labelled by SigR1 (**g**) and p62 (**h**) antibody (merge image for GFP-LC3, green, and SigR1/p62, red). Note the localization of SigR1 at the periphery of this particular cell (**g**, arrowheads) and the co-localization of GFP-LC3 with globular p62 accumulations. Scale bar, 10 *μ*m. (**i**) Co-localization of SigR1 with p62 and LC3 in E102Q-SigR1 fALS lymphoblastoid cells compared to healthy control lymphoblastoid cells. (**j**) Cyto-ID (green) staining (right) showing the accumulation of autophagosomes in E102Q-SigR1 fALS lymphoblastoid cells. (**k**) Immunoblot analysis of established autophagy markers in E102Q-SigR1 fALS lymphoblastoid cells in comparison to healthy controls. (**l**) A431 cells were transfected as described above. Forty-eight  hours later, transfected cells were processed for the EGFR degradation assay as described in Materials and Methods and analyzed by immunoblotting with the EGFR antibody. Note the delayed EGFR degradation in mSigR1 expressing cells. (lower) Quantification of immunoblot analysis. Values are expressed as mean±S.D. from three independent experiments. *P<0.05*.* (**m**) NIH3T3 cells expressing RFP-GFP-LC3 were transfected with pcDNA, wtSigR1 or mSigR1. Forty-eight hours later the fusion of autophagosomes with lysosomes was measured by live cell imaging. Scale bar, 25 *μ*m. (lower) The rate of autophagosome maturation reflected by the Pearson coefficient (green/red fluorescence ratio) at each time point indicated. Values are represented as means±S.E.M. of triplicate experiments *P<<0.0001. (**n**) EM picture of NIH-3T3 cells stably expressing RFP-GFP-LC3 transfected with mSigR1. Note the autophagosome (white arrow) and lysosomes (gray arrow) without any fusion

Transfection of mSigR1 in A431 and MCF-7 cells resulted in increased accumulation of p62 and LC3II (Figure 5a and Supplementary Figure 4A) together with epidermal growth factor receptor (EGFR) and LAMP-1 (Figure 5a). Consistent with the immunoblot results, immunofluorescence (Figure 5b) and CYTO-ID dye labelling (Figure 5c) showed globular LC3 accumulation co-localized with SigR1 aggregates (arrows) in mSigR1-transfected cells. Similarly, p62 was accumulated and abnormally associated with SigR1 aggregates (Figure 5d). Moreover, in nutrient-starved A431 cells (to induce autophagy), expression of mSigR1 increased the stability of p62 and LC3II levels (Supplementary Figure 4B). Furthermore, autophagy induction (Rapamycin) or inhibition (Bafilomycin A) only marginally, if at all, affected p62 and LC3II levels in mSigR1-expressing MCF-7 cells compared to wtSigR1 controls (Supplementary Figure 4C), suggesting an impairment of autophagic degradation.

mSigR1 expression in mouse fibroblasts (NIH-3T3) stably expressing GFP-LC3^14^ led to aggregation and abnormal accumulation of GFP-LC3 (Figure 5e). Absence of a significant increase in LC3II and p62 levels even after Bafilomycin-A treatment also suggested abnormal autophagy (Figure 5f). In primary MEFs isolated from GFP-LC3 tg mice^22^ that we transfected with wtSigR1, normal ER-associated SigR1 staining and few GFP-LC3 puncta together with very few p62-positive puncta (Figures 5g and h) were observed. In contrast, mSigR1-transfected MEFs showed sub-plasmalemmal accumulation of mSigR1 (Figure 5g) together with significant globular accumulation and co-localization of GFP-LC3 and p62 (Figure 5h). These findings were corroborated by immunoblot analyses (Supplementary Figure 4D). Finally E102Q-SigR1 PLCs showed similar aggregation and co-localization of p62 and LC3II together with mSigR1 aggregates (Figure 5i), but no co-localization of LAMP1 and SigR1 aggregates (Supplementary Figure 4F). These findings were strengthened by Cyto-ID-LC3 labelling yielding large globular accumulations of LC3 in E102Q-SigR1 PLCs (Figure 5j) and by p62, LC3 and LAMP1 immunoblots (Figure 5k).

Next, we determined which steps within the autophagic processing chain (internalization, fusion or lysosomal degradation) were impaired by mSigR1. Over-expression of mSigR1 in A431 cells did not impair receptor internalization after 10 min of EGF stimulation compared to wtSigR1 (Supplementary Figure 4E). Activated EGFR was efficiently degraded after internalization in control cells (pcDNA and wtSigR1), however EGFR in mSigR1-expressing cells were only slightly reduced (Figure 5l, quantification below). Live cell imaging of a NIH-3T3 reporter cell line (see Methods and refs 14, 23) showed significant impairment of autophagosomes-lysosome fusion in mSigR1-expressing cells as indicated by the minimal loss of GFP fluorescence and maximal co-localization of RFP and GFP signals (Figure 5m) compared to controls. Accordingly, EM revealed accumulation of double membrane-bound autophagosomes (AV) not fusing with lysosomes (Figure 5n). Altogether, our findings suggest that mSigR1 aggregates impair the autophagic degradation and clearance of autophagosomes.

### mSigR1 impairs vesicular transport

**Figure 6:**
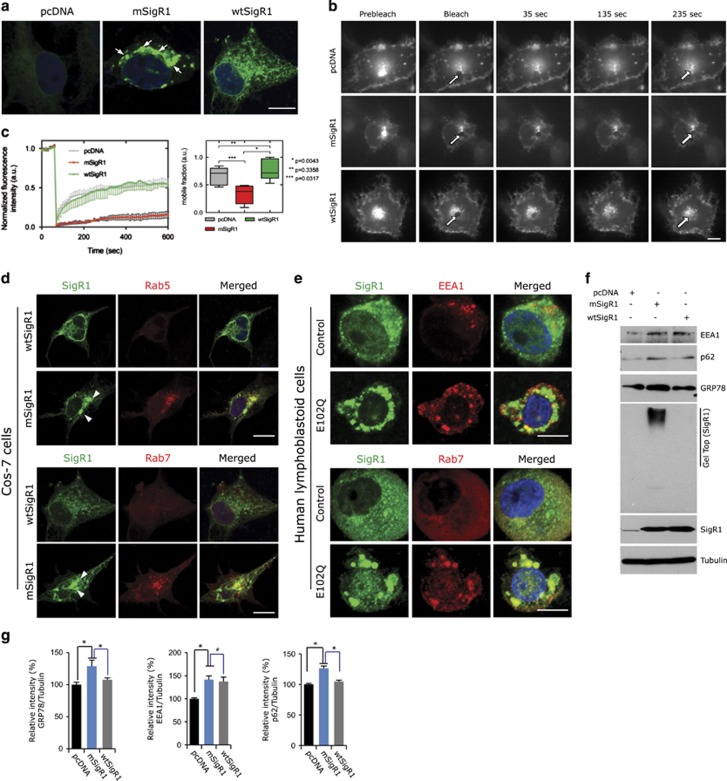
mSigR1 impairs ER to Golgi transport. (**a**) Cos7 cells showing abnormal SigR1 accumulations (arrows) after mSigR1 transfection and normal ER localization of SigR1 (right) after wtSigR1 transfection. Scale bar, 15 *μ*m. (**b**) Cos7 cells were co-transfected with VSVG-GFP together with pcDNA, wtSigR1 or mSigR1. 48 h after transfection, fluorescence associated with the Golgi complex was photobleached (FRAP, see Materials and Methods) with a high-intensity laser beam. Subsequently, the inward delivery of VSVG-GFP from pre-Golgi intermediates was monitored for the indicated periods of time. Scale bar, 10 *μ*m. (**c**) Fluorescence recoveries after photobleaching curves of mSigR1-transfected cells show a clear decrease of the mobile fraction as compared to cell transfected with pcDNA or wtSigR1. Error bars indicate the S.E.M.; (right) the comparison of the mobile fractions in pcDNA, wtSigR1 and mSigR1 expressing cells. In the box plots, the line in the middle of the box indicates the median; the top line indicates the 75th quartile, whereas the bottom line indicates the 25th quartile. Whiskers represent the 10th and 90th (upper) percentile, respectively. (**d**) mSigR1 aggregates (arrowheads) co-localize with the endosomal markers Rab5 (upper) and Rab7 (lower) in Cos-7 cells transfected with wtSigR1 and mSigR1, respectively. Scale bar, 10 *μ*m. (**e**) Co-localization of mSigR1 aggregates with the endosomal markers EEA1 and Rab7 in E102Q-SigR1 fALS patients' lymphoblastoid cells compared to healthy controls. Scale bars, 15 *μ*m. (**f**) Immunoblot analysis of Cos-7 cells transfected with pcDNA, wtSigR1 and mSigR1. (**g**) Quantification of band intensities normalized against *α*-tubulin depicted in (**f**). Values are expressed as mean±S.D. from three independent experiments. *P<0.05

mSigR1 forms ER-associated aggregates (Figures 1a and 6a) accompanied by disturbed ER ultrastructure (Figures 4a and b) and abnormal Golgi morphology (Figures 1g and 2k). Therefore, ER/Golgi defects might affect vesicular transport from ER to Golgi and *vice versa*. Thus we analyzed VSVG-GFP fluorescence recovery after photobleaching (FRAP) imaging.^24, 25^ In control COS-7 cells co-transfected with VSVG-GFP and either pcDNA or wtSigR1, VSVG-GFP vesicle trafficking was efficient as indicated by fast VSVG-GFP recovery (Figures 6b and c and Supplementary Movie 1). In contrast, in mSigR1-expressing cells intracellular movement of VSVG-GFP vesicles was severely impaired (Figures 6b and c and Supplementary Movie 1). Consistent with this mSigR1 co-aggregated with the endosomal markers Rab5 and Rab7 (Figure 6d) and early endosomal antigen 1 (EEA1) was increased together with ER stress and autophagy markers in immunoblots from mSigR1-transfected COS-7 cells (Figures 6f and g). Recapitulating the known effect of over-expression of other ER membrane proteins,^26^ transfection of wtSigR1 mildly affected EEA1 and LC3II, but not p62 and GRP78. E102Q-SigR1 PLCs also showed similar co-aggregation of early (EEA1) and late (Rab7) endosomal markers with mSigR1 (Figure 6e). Our results are consistent with a recent study showing reduced intracellular mobility of mSigR1,^27^ with previous reports describing that VSVG transport is impaired by ER stress,^24, 25^ and also with our own previous work showing ultrastructural abnormalities of the ER/Golgi complex in SigR1-deficient cells.^11, 14^

### mSigR1 accumulation leads to altered distribution of RBPs

**Figure 7:**
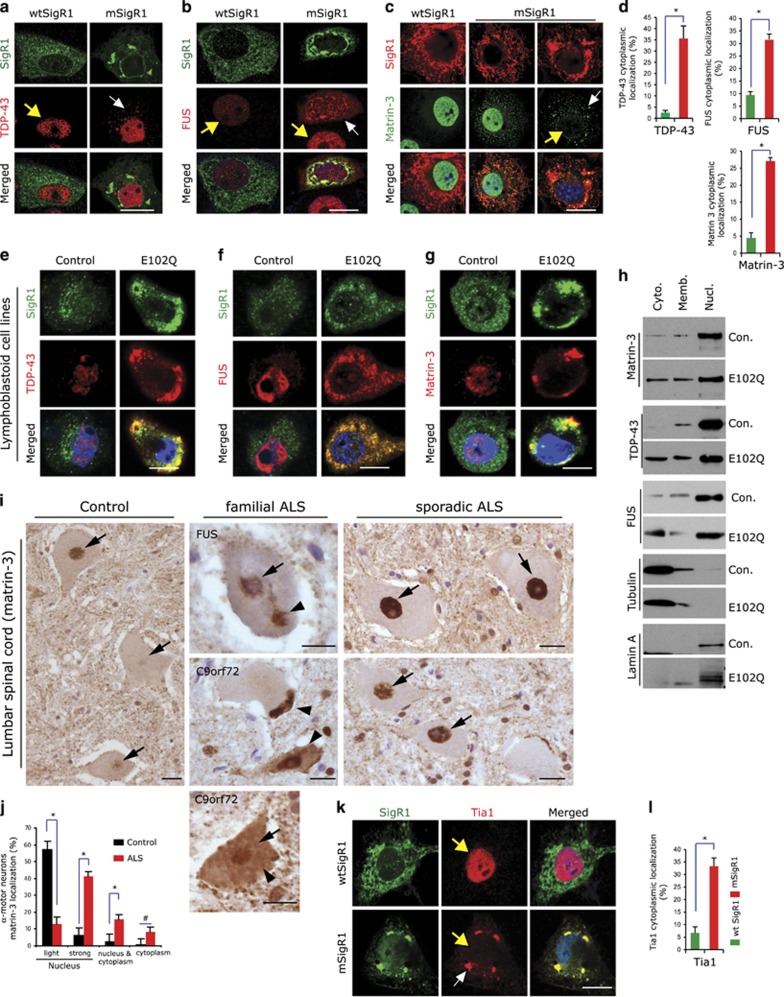
mSigR1 accumulation leads to altered RNA-binding protein homeostasis. (**a**) Co-staining of SigR1 and TDP-43 in MCF-7 cells transfected with either wtSigR1 or mSigR1. Note the minor cytoplasmic TDP-43 accumulations (white arrow) without co-localization with SigR1, in contrast to the more pronounced TDP-43 aggregates co-localizing with SigR1 in E102Q-SigR1 fALS lymphoblastoid cell depicted in **e**. Scale bar, 15 *μ*m. (**b**) Translocation of FUS from the nucleus (yellow arrow) to the cytoplasm (white arrow) and co-localization with SigR1 aggregates in MCF-7 cells expressing mSigR1 compared to wtSigR1-transfected cells. Scale bar, 10 *μ*m. (**c**) Immunofluorescence staining of SigR1 and matrin-3 in MCF-7 cells expressing wtSigR1 or mSigR1. Note the cytoplasmic accumulations (white arrow) along with the loss of nuclear matrin-3 (yellow arrow) corresponding to the higher amount of globular mSigR1 aggregates. Scale bar, 10 *μ*m. (**d**) Quantification (*n*=3 each) of the experiments illustrated in (**a**–**c**). *P<0.05. (**e**–**g**) Nuclear loss of TDP43 (**e**)**,** FUS (**f**) and matrin-3 (**g**) and their co-localization with mSigR1 aggregates in E102Q-SigR1 fALS lymphoblastoid cells compared to healthy controls. Scale bar, 10 *μ*m. (**h**) Immunoblot analysis of subcellular fractions obtained from E102Q-SigR1 patients' lymphoblastoid cell lines compared to healthy controls (*n*=2 for fALS; *n*=3 for control). Matrin-3, FUS and TDP-43 distributions are shown in the cytoplasmic (Cyto.), membrane (Memb.) and nuclear (Nucl.) fractions. Note the translocation of matrin-3 from the nucleus to the cytoplasm in E102Q-SigR1 patients' lymphoblastoid cells. Tubulin is used as a loading control and Lamin A as a positive control for the nuclear fraction. (**i**) Immunohistochemical analysis of lumbar *α*-MNs using matrin-3 antibody in sALS and fALS compared to normal controls. Cytoplasmic accumulation (arrowheads, middle) of matrin-3 in *α*-MNs of fALS patients harbouring FUS and C9ORF72 mutations. Strong nuclear immunoreactivity of matrin-3 (arrows) is evident in sALS *α*-MNs (right), whereas the control shows an overall weaker nuclear matrin-3 signal (arrows, left). Paraffin sections, DAB-immunohistochemistry; scale bars, 20 *μ*m. (**j**) Quantification of immunohistochemical data illustrated in **I**; for the immunohistochemical analysis *n*=12 sALS; *n*=8 (C9ORF72); *n*=4 (FUS) and *n*=4 control cases were examined. *P<0.05, while # denotes absence of significance (**k**) loss of nuclear Tia1 (yellow arrows) and accumulation of cytoplasmic Tia-1 (white arrow in the middle panel) positive stress granules co-localized with mSigR1 aggregates in MCF-7 cells. Scale bar, 15 *μ*m. (**l**) Quantification of data shown in (**k**). *P<0.05

RBPs are multifunctional and play a major role in sALS pathophysiology.^28^ Alterations of RBPs homeostasis can directly or indirectly modulate protein quality control.^29^ Cytoplasmic aggregates along with granular cytoplasmic staining for endogenous TDP-43 was found in mSigR1-transfected MCF-7 cells, but not in controls (Figure 7a). Similarly cytoplasmic translocation and aggregation of FUS was observed (Figures 7b and d). MEF-GFP-LC3 cells expressing mSigR1 showed increased cytoplasmic staining for endogenous TDP-43 together with increased accumulation of GFP-LC3 (Supplementary Figure 4H).

Mutations in the nuclear matrix protein matrin-3 can cause fALS^30^ and autosomal dominant distal myopathy with vocal cord and pharyngeal weakness;^31, 32^ matrin-3 interacts with TDP-43, FUS and other RBPs.^33^ Matrin-3 also showed cytoplasmic mis-localization in MCF 7 cells expressing mSigR1 (Figures 7c and d). Occasionally in some cells having large globular, compact mSigR1 aggregates, matrin-3 was found exclusively in the cytoplasm with complete loss of nuclear signal (Figure 7c). Immunoblot analysis of subcellular fractions of MCF-7 cells confirmed these observations (Supplementary Figure 4G). Confocal imaging of E102Q-SigR1 PLCs confirmed that aggregates of mSigR1 were co-localized with TDP-43 (Figure 7e), FUS (Figure 7f) and matrin-3 (Figure 7g). Immunoblots of the subcellular fractions of E102Q-SigR1 PLCs confirmed increased cytoplasmic matrin-3, TDP43 and FUS (Figure 7h). Consistent with these findings immunohistochemistry of the lumbar spinal cord of fALS patients harbouring *FUS* and *C9orf72* mutations revealed cytoplasmic matrin-3 accumulations in *α*-MNs (Figures 7i and j). By double-immunofluorescence these accumulations did not co-localize with pTDP-43 aggregates (Supplementary Figures 5A and B) consistent with prior observations.^30^ We also confirmed the finding by Johnson *et al.*^30^ that lumbar *α*-MNs of sALS and fALS patients show increased nuclear matrin-3 immunoreactivity. Interestingly, however, in the E102Q-SigR1 transfected cells and in the E102Q-SigR1 PLCs (Figures 7c and g) a high degree of cytoplasmic SigR1 accumulation was not only associated with increased cytoplasmic matrin-3 immunoreactivity, but also accompanied by a loss of nuclear matrin-3 staining. These data indicate that cytoplasmic mis-localization of matrin-3 in sALS and fALS MNs and in E102Q-SigR1 expressing cells might be pathophysiologically relevant.

Aggregation of these RBPs proceeds through the stress granule pathway (see above and refs 5, 34). Consistent with the cytoplasmic mis-localization of several RBPs, MCF-7 cells expressing mSigR1 showed prominent Tia 1-positive stress granules co-localized with SigR1 aggregates (Figures 7k and l) and cytoplasmic P-body formation in both E102Q-SigR1 PLCs and transfected MCF-7 cells (Supplementary Figure 4I and J). However SigR1 aggregates did not exactly co-localize with adjacent P bodies (Supplementary Figures 4I and J). These results indicate that mSigR1 expression and aggregation leads to abnormal RNA-binding protein homeostasis and stress granule formation.

### E102Q-SigR1 mediated ALS pathogenesis

**Figure 8:**
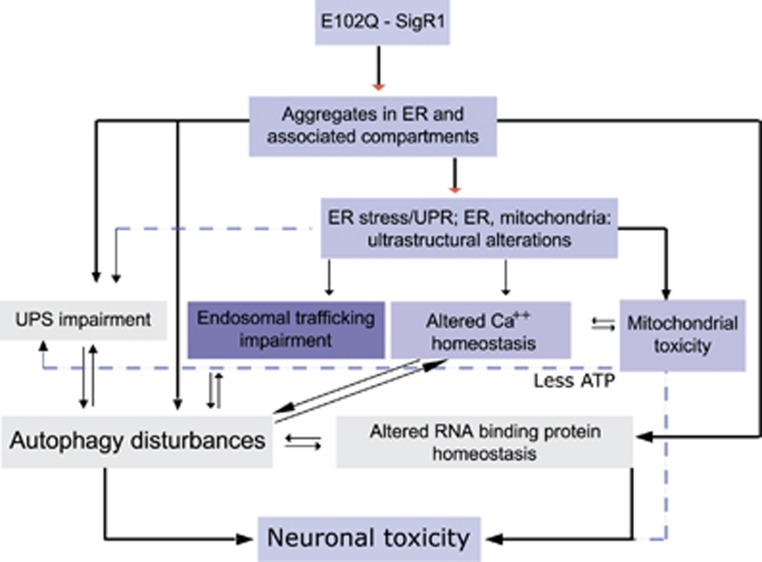
Illustration showing the proposed mechanisms of ALS pathogenesis associated with the E102Q mutation in SigR1

Schematic representation (Figure 8) of the interdependent pathophysiological mechanisms of ALS pathogenesis associated with the E102Q mutation in SigR1.

## Discussion

Mutations in several ER proteins, including SigR1, cause MNDs. For instance, the P56S mutation in the *VAPB* gene leads to a form of fALS, ALS-8,^35, 36^ characterized by distinct ultrastructural ER alterations and defective protein degradation pathways.^37^ Similarly, mutations in ER chaperones such as SIL1, HSPB8 and HSJ1 lead to familial neurodegenerative disorders including MNDs.^38, 39, 40^ ER (co-) chaperones including SigR1 and SIL1 accumulate in surviving MNs in sALS and might serve protective functions.^11, 41^ E102Q-SigR1-associated disease shows an autosomal recessive inheritance pattern suggesting a ‘loss-of-function’ pathomechanism consistent with a recent report^42^ and also with our previous reports.^11, 14^ However, neither the E102Q nor the recently found homozygous (E138Q and E150K) SigR1 mutations^9^ could be linked to transcriptional silencing or defective translation so far.

### ER stress and structural alterations of the ER/nuclear envelope

ATF4 is required for the activation of SigR1 transcription and upregulation of SigR1 suppresses ER stress-mediated cell death, thus considered to be neuroprotective.^43^ Consistent with this, Gregianin *et al.*^9^ recently showed that over-expression of wtSigR1 suppressed ER stress-induced mitochondrial injury. We found that mSigR1 promotes prolonged ER stress (Figures 1j and l and 2d and g) and altered ER/Golgi morphology (Figure 4) followed by defective endosomal trafficking (Figures 6b and c). We previously observed ER stress and defects in endosomal trafficking after SigR1 protein depletion.^11, 14^ Moreover, E102Q-SigR1 was recently shown to lose its ability to bind to the MAM, suggesting yet another loss of function pathomechanism.^9^ Evidence favoring an additional toxic gain of function results from very recent findings suggesting reduced mobility of E102Q-SigR1.^27^ Analogously, the wtVAPB protein is involved in UPR and P56S-mutated VAPB causes ALS-8,^35, 36^ forms ER-associated aggregates, alters ER/Golgi ultrastructure, protein quality control^37, 44^ and endosomal trafficking^45, 46^ and causes ER stress-related cell death *in vitro*^44^ and motor abnormalities in P56S-VAPB transgenic mice.^37^ Loss of SIL1, another ER (co-)chaperone, affects ER homeostasis in mouse models;^47^ loss of a single functional SIL1 allele in the G93A-SOD ALS mice enhanced ER stress and exacerbated ALS pathology.^41^ These results are reminiscent of the phenotypes in SigR1^−/^^−^ mice.^13^ Conversely, AAV delivery of SIL1 to fALS MNs restored ER homeostasis, delayed muscle denervation and prolonged survival^41^ similar to the improved motor function and MN survival in SOD1 fALS mice after treatment with a SigR1 agonist.^15^

SigR1 mainly resides at the ER-MAM interface; upon agonist-mediated activation SigR1 translocates from the ER to the nuclear envelope where it binds to emerin and recruits chromatin-remodeling molecules including lamin A/C.^48^ Knockdown of SigR1 protein induces disruption of the nuclear envelope complex.^48^ In line with these observations, we observed reduced emerin staining of the nuclear envelope in E102Q-SigR1-transfected cells (Figure 1b) and E102Q-SigR1 PLCs (Figure 2a). In addition, we found aberrant emerin co-localization with mSigR1 aggregates indicative of an additional toxic gain of function (Figure 1c,Figure 2a). Interestingly, SIL1-deficient mice and human Marinesco Sjögren syndrome (MSS) patients lacking SIL1 also display myonuclear envelope defects.^47^ Consistent with this P56S-VAPB affects the retrograde trafficking of ERGIC-53^49^ and shuttling of emerin from the ER to the inner nuclear membrane,^49^ and knockdown of endogenous VAPB actually recapitulates this phenotype.^49^ Thus, nuclear envelope defects emerge as a common denominator of pathologies associated with the ALS proteins SigR1, VAPB and SIL1.

### MAM, mitochondrial impairment and intracellular calcium dysregulation

Deregulation of neuronal intracellular Ca^2+^ signaling is a major pathomechanism observed in many neurodegenerative disorders such as AD, PD, ALS and HD (reviewed in ref. 50). Besides its chaperone function SigR1 also regulates IP3-mediated Ca^2+^ homeostasis at the MAM^6^ and depletion of SigR1 disturbs Ca^2+^ homeostasis^11, 14^ and ER-mitochondria crosstalk.^13^ MAM alterations are involved not only in ALS but also in AD, PD and in several axonopathies such as hereditary spastic paraplegia and axonal Charcot–Marie–Tooth disease (reviewed in ref. 51). In the present study, mSigR1 expression led to MAM alterations and impaired SOCE and failed to trigger efficient Ca^2+^ release from the ER through IP3R (Figures 3a–c). These calcium handling and MAM defects are most likely caused by both the observed disruption of ER/mitochondrial structure and by alterations in STIM1-mediated Ca^2+^ influx because of lowered levels of STIM1 in the E102Q-SigR1-expressing cells (Figures 3d and e). These findings are in line with our previous studies^11, 14^ and also with the recent study by Gregianin *et al.* describing the deleterious effect of two new mutations in SigR1 (E138Q and E150K) on cell viability due to an altered MAM and impaired global Ca^2+^ signalling.^9^ Interestingly, another study (by Tagashira *et al.*^17^) using transient expression of E102Q-SigR1 showed aberrant mitochondrial Ca^2+^ uptake and ATP production,^17^ consistent with our results. However, both groups studied only IP3R-mediated Ca^2+^ homeostasis and its effects on mitochondria. In contrast, we also examined STIM1-induced SOCE as an alternate mechanism causing globally altered Ca^2+^ homeostasis.

### Proteostasis (UPS/autophagy) and RBPs

The turnover of TDP-43 and FUS and of other RNA stress granule proteins is regulated by UPS and autophagy;^52, 53^ chemically augmenting autophagic flux leads to the clearance of abnormal aggregates of these proteins.^52, 53^ Furthermore, alterations of mRNA biogenesis/processing can modulate protein expression under various cellular stress responses.^29^ We found that mSigR1 aggregation leads to ER stress (Figures 1 and 2), autophagy impairment (Figure 5) and aberrant extra-nuclear localization and aggregation of the RBPs TDP-43, FUS and matrin-3 (Figures 7a–h). Matrin-3 regulates transcription and stability of several RBPs including TDP-43 and FUS;^33^ *MATR3* mutations cause ALS and distal myopathy.^30, 31, 32^ Recently, mice over-expressing human matrin-3 were reported to develop muscular atrophy and altered spinal cord distribution of matrin-3 protein.^54^ Consistent with previous reports^30, 31, 32^ on human matrinopathy, we observed both cytoplasmic and nuclear matrin-3 accumulation in E102Q-SigR1 over-expressing cells, along with the aggregation of other RBPs relevant to ALS (TDP-43 and FUS). Furthermore, matrin-3 mis-localization was induced by misfolded protein stress and impairment of degradation pathways in mSigR1 expressing cells (Supplementary Figure 5C). Interestingly, transfected cells showing large cytoplasmic accumulations of SigR1 also showed increased cytoplasmic matrin-3 immunoreactivity suggesting that the E102Q-SigR1 mutation leads to a toxic gain of function involving matrin-3 (Figures 7c and g). These results are consistent with a previous report demonstrating cytoplasmic accumulation of matrin-3 in *α*-MNs of ALS patients harboring *C9ORF72* and *MATR3* mutations. Here, we confirm the increased cytoplasmic matrin-3 immunoreactivity in *C9ORF72* fALS *α*-MNs and demonstrate that cytoplasmic matrin-3 aggregation also occurs in lumbar *α*-MNs of fALS patients harboring *FUS* mutations. Irrespective of the presence or absence of cytoplasmic matrin-3 aggregation, we also confirm the observation that nuclear matrin-3 staining is increased in sALS and fALS lumbar *α*-MNs.^30^ However, unlike mTDP-43 and mFUS, cytoplasmic accumulations of matrin-3 in fALS *α*-motor neurons are rare.^30^ Taken together, our immunohistochemical data from human ALS autopsy cases are in line with the above described results obtained with E102Q-SigR1 over-expressing cultured cells. Our data indicate that cytoplasmic mis-localization of matrin-3 together with other RBPs in sALS and fALS cells as well as in E102Q-SigR1 expressing cells might be pathophysiologically relevant.

With regard to TDP-43 redistribution in E102Q-SigR1 transfected cells, our data confirm results of a recent study^17^ describing TDP-43 mis-localization along with deranged mitochondrial ATP production and proteasome activity as a consequence of mSigR1 over-expression. Our observations are also in accordance with studies examining a causal relationship of other ER proteins like Sil1 and VAPB with TDP-43 proteinopathy. P56S-VAPB, for instance, potentiated the TDP-43-induced MN death, whereas wt-VAPB had opposite effects.^45^

We observed that both the ER stressor thapsigargin and the autophagy inhibitor bafilomycin A led to similar extra-nuclear localization of TDP-43 and matrin-3 in NSC-34 (motor neuron-like) cells; induction of autophagy either by rapamycin or by the SigR1 agonist PRE084 prevented this mis-localization (Supplementary Figures 5C and D). SigR1 agonists such as PRE084 have already been shown to reduce protein aggregation and ER stress^11^ and to be neuroprotective.^15^ Thus, our data suggest that the ALS proteins SigR1, TDP-43 and matrin-3 are interdependent in regulating cellular protein quality control pathways and that dysregulation of the RBPs matrin-3, FUS and TDP-43 can be directly caused by mSigR1 aggregation. In addition, indirect mechanisms such as autophagy impairment due to mSigR1 expression could be active, supporting a simultaneous toxic gain and loss of function of mSigR1 (summarized in Figure 8), similar to the recently described pathomechanism (s) related to mutated TDP-43, C9orf72, FUS,^46^ SOD1 (ref. 55) and VAPB.^44, 49^

## Materials and methods

### Reagents

Fluorescent nucleic acid stain Hoechst 33258 was purchased from Molecular Probes (Eugene, OR, USA). Thapsigargin,Tunicamycin, EGF, Rapamycin, Bafilomycin A, Fura 2AM, NaCl, KCl, pluronic acid, CaCl2, MgCl2, glucose, HEPES 4-(2-hydroxyethyl)-1-piperazineethanesulfonic acid, *N*-acetyl-Asp-Glu-Val-Asp-7 amido-4-methylcoumarin and Bradykinin were purchased from Sigma Aldrich (Munich, Germany). Suc-Leu-Leu-Val-Tyr-AMC was received from Enzo (Lausen, Switzerland). Alamar Blue was purchased from Invitrogen, Thermo Fischer Scientific, Carlsbad, CA, USA. The CYTO-ID Autophagy detection kit was purchased from Enzo.

### Antibodies

The antibodies used in this study and their dilutions are described in Supplementary Table 1.

### Cell culture, transient transfection and treatments

#### Cell culture and treatment

Human epithelial cancer cells (HeLa), African green monkey kidney cells (COS-7), human breast cancer cells (MCF-7) and NSC34 motor neuron-like cells were cultured in Dulbecco's modified Eagle's medium (DMEM, Invitrogen), supplemented with 10% FBS and 1% antibiotic/anti-mycotic solution (Invitrogen). Mouse embryonic fibroblasts (MEF) obtained from the GFP-LC3 transgenic mice^22^ were also maintained in DMEM supplemented with 10% FBS and 1% antibiotic/anti-mycotic solution. The human epidermoid carcinoma cell line A431 was grown in Dulbecco's modified Eagle's medium (DMEM, Invitrogen) supplemented with 10% FBS and 0.1% Gentamycin. NIH-3T3 cells stably expressing GFP-LC3 or tandem mCherry-EGFP-LC3 were cultured in DMEM supplemented with 10% FBS, 1% penicillin/streptomycin and puromycin (Sigma Aldrich). Lymphoblasts obtained from E102Q-SigR1 fALS patients and healthy controls were cultured in RPMI 1640 medium containing 20% FBS, 1% penicillin/streptomycin, 1% L-glutamin and 0.056% amphotericin. Cells were maintained in a humidified incubator at 37 °C and 5% CO_2_. Generation of NIH-3T3 cells stably expressing GFP-LC3 or tandem mCherry-EGFP-LC3 with retroviral infection is described elsewhere.^14^

### Plasmids and transfection

In order to investigate the molecular mechanisms of E102Q-SigR1-related pathogenesis, cells were transfected to express either wtSigR1 or mSigR1. The vectors for the ectopic expression of wtSigR1 or E102Q-SigR1 have been described already.^7^ The empty pcDNA vector was used as a transfection control. All cell lines were transfected using Lipofectamine 2000 reagent (Invitrogen) according to the manufacturer’s recommendations. After 4h incubation at 37 °C and 5% CO_2_ the transfection reagent containing medium was replaced by fresh medium and analysis was performed 48h later. VSVG-GFP used for vesicle transport analysis was a kind gift of Prof. Jennifer Lippincott-Schwartz. For proteasome activity analysis, cells were co-transfected with either pcDNA, wtSigR1 or mSigR1.

### Post-mortem tissues and Immunohistochemistry

Paraffin sections of human post-mortem lumbar spinal cord (*n*=6 age-matched controls, *n*=15 sALS, *n*= 9 C9orf72, *n*= 4 FUS; ALS patients) were obtained from the Amsterdam Academic Medical Center (AMC), Division of neuropathology, Department of Pathology ALS Bank (Amsterdam, The Netherlands) Research Code provided by the Medical Ethics Committee of the AMC (Amsterdam, The Netherlands; approved protocol: W11_073). The post-mortem tissues were obtained within 6–12 h after death. All tissues were used in compliance with the Declaration of Helsinki.

Immunohistochemistry was performed as previously described^56^ on paraffin sections (5–6 *μ*m thickness), de-waxed, rehydrated and heated in Citrate Buffer (pH 6.0), (Thermo Scientific concentrate cat.no.- 005000) for antigen retrieval. Sections were then incubated with primary antibody (Supplementary Table 1) for 1 h at room temperature. After washing in 0.1M PBS, sections were incubated with appropriate HRP-conjugated secondary antibodies (1:200, Immunologic, Duiven, The Netherlands) for 1 h, followed by 3,3-diaminobenzidine (DAB) visualization (Immunologic). For double immunofluorescence, secondary antibodies conjugated with Alexa fluor 594 and Alexa-fluor 488 were used (Invitrogen), followed by cover slipping with mounting medium containing diamidinophenylindole (DAPI) for nuclear counterstain. Immunoperoxidase stained sections were visualized and photographed using a Zeiss Axioplan microscope with a Zeiss Axiocam HR camera (Zeiss, Oberkochen, Germany) and immunofluorescence was visualized using a Zeiss LSM 700 confocal microscope (Zeiss) and images processed using Zeiss LSM software and Adobe Photoshop CS5.

### Immunocytochemistry

HeLa, MCF-7, Cos-7, MEF and NIH-3T3 cells were cultured on *μ*-dishes (ibidi, GmbH, Planegg/Martinsried, Germany) and transiently transfected either with wtSigR1 or mSigR1. After 48 h cells were fixed in 4% PFA and processed for confocal microscopy. Permeabilization with 0.5% Triton X100 and blocking with 4% skimmed milk/goat serum was followed by primary antibody incubation overnight at 4 °C. Secondary Alexa488- or Alexa594-conjugated anti-mouse or anti-rabbit antibodies (Invitrogen) were used for visualization. Nuclei were stained with Hoechst 33342 (1 *μ*g/ml) or were mounted with DAPI containing fluorescent mounting media (DAKO) and visualized using a Zeiss LSM 700 confocal microscope. Resulting images were processed using the Zeiss LSM software and Adobe Photoshop CS5 (Adobe Systems, San Jose, CA, USA).

### Fura-2 calcium imaging

The intracellular calcium ion concentration, [Ca^2+^]i, was measured using a conventional Fura-2 technique as previously described.^11^ After 48 h of transfection with pcDNA, wtSigR1 or mSigR1, NSC-34 and MCF-7 cells cultured in glass bottom *μ*-dishes (ibidi), were loaded with the membrane-permeable AM-form of Fura-2 (1.5 ng/*μ*l; Invitrogen) in the presence of pluronic acid (25 %) for 30 min at 37 °C. Emitted fluorescence at 530 nm (detected using a PCO (Sensicam: pco.imaging), Kelheim, Germany) in response to alternate excitation at 340 nm and 380 nm (using the Polychrome V monochromator; TILL Photonics, Gräfelfing, Germany) was used to measure intracellular Ca^2+^ concentrations. Data were shown as emission ratios in response to 340 nm /380 nm excitation. Whole-cell calcium measurements of NSC-34 and MCF-7 cells transfected with pcDNA, wtSigR1 or mSigR1 were obtained at room temperature (23–25 °C). During the imaging procedure, cells were kept in a bathing solution containing 100 mM NaCl, 5.4 mM KCl, 2 mM CaCl2, 1 mM MgCl2, 10 mM HEPES, 10 MES, 5.5 glucose and pH was adjusted to 7.4.

### ER stress assay

To investigate the ER stress levels in pcDNA, wtSigR1 and mSigR1 expressing cells, we used the ERSE reporter system (Cignal reporter assay kit, Qiagen Cat. No.–CCS-2032L, Hilden, Germany) in combination with the dual luciferase system (Promega Cat.No. – E1910, Madison, WI, USA) according to the manufacturers' protocols. Cells were co-transfected with pcDNA, wtSigR1 and mSigR1 together with the ERSE reporter construct containing the luciferase gene. After 24 h of incubation, luciferase activity was measured by using the dual luciferase assay for firefly and renilla luciferase detection.

### Mitochondrial toxicity assay

Mitochondrial toxicity exerted by mSigR1 was measured using the mitochondrial ToxGlo assay kit (Promega: Cat. No–G8000) according to the manufacturer's protocol. MCF-7 cells were plated on a 96 well plate and transfected with wtSigR1 or mSigR1. 48 h after transfection, cells were incubated with cytotoxicity reagent at 37 °C for 30 min and fluorescence was measured at 520 nm.This value represents the membrane integrity (MI). Thereafter the same plate was equilibrated at room temperature and then incubated with the ATP detection substrate; luminescence was measured after 5 min–1 h for the detection of ATP production.

### Transmission electron microscopy (TEM)

MCF-7 and NSC-34 cells were transfected with pcDNA, wtSigR1 or mSigR1 as described above. Cells were collected by scraping, suspended lymphoblast cultures from healthy controls and ALS patients were collected by brief centrifugation (5000 r.p.m. for 5 min) and then washed in 0.1 M phosphate buffer and immediately fixed with 2.5% glutaraldehyde in 0.1 M phosphate buffer for 24 h followed by washing in buffer for another 24 h. Cell pellets were collected by centrifugation (1000 r.p.m., 5 min) and embedded in 2% agarose (at 60 °C; Fluka #05073). Small blocks of embedded cells were sliced and post-fixed in 2.5% glutaraldehyde for 24 h followed by washing in 0.1 M phosphate buffer for 24 h. Agarose blocks were then incubated in 1% OsO4 (in 0.2 M phosphate buffer) for 3 h, washed twice in distilled water and dehydrated with using ascending alcohol concentrations (i.e., 25, 35, 50, 70, 85, 95 and 100% each step for 5 min). Dehydrated blocks were incubated in propylenoxide followed by subsequent 20 min incubation in a 1:1 mixture of epon (47.5% glycidether, 26.5% dodenylsuccinic acid anhydride, 24.5% methylnadic anhydride and 1.5% Tris (dimethylaminomethyl) phenol) and propylenoxide. The samples were then incubated in epoxy resin for 1 h at room temperature followed by polymerization (28 °C for 8 h, 80 °C for 2.5 h and finally at room temperature for 4 h). Ultra-thin sections (70 nm) were mounted on grids for electron microscopy and examined using a Philips CM10 transmission electron microscope (Philips, Amsterdam, The Netherlands) as already described.^11^

### Fluorescence recovery after photobleaching (FRAP)

For live cell imaging experiments, Cos7 cells were seeded on glass-bottomed dishes (ø 6 cm) and co-transfected either with pcDNA/VSVGts045-GFP, wtSigR1/VSVGts045-GFP or mSigR1/ VSVGts045-GFP. VSVGts045-GFP was allowed to accumulate in the ER at 42 °C for 8 h and cells were imaged on an Axio Observer Z1 inverted microscope equipped with heating stage and CO_2_ controller (Zeiss) maintained at a constant temperature of 32 °C. A portion of the ER (ø ~3.84*μ*m) in the periphery of the cell was photobleached using a 405 nm laser driven by the UGA-40 control unit (Rapp Opto Electronic GmbH, Wedel, Germany). The recovery of the fluorescent signal was monitored by imaging the cells every second for 15 min. Imaging was done using an Evolve EM-CCD camera driven by ZEN software (Zeiss). For all experiments the bleached area and the duration of the laser impulse were kept constant. The extent of recovery of the fluorescent signal was determined using ImageJ to measure the average pixel intensity values within three distinct regions of interest: ROI1: bleached area, ROI2: unbleached area within the cell and ROI3: background. Normalized FRAP recovery curves and the mobile fraction were calculated using the program easy FRAP.

### Live cell imaging to analyze RFP-GFP-LC3 fusion

To analyze the dynamics of the RFP-GFP-LC3 fusion protein, GFP and RFP channels were acquired every minute for up to 4 h using the imaging system described above. The extent of autophagosome maturation was determined by measuring the co-localization of the GFP and RFP signals as expressed by the Pearson’s coefficient using ZEN software.

### Immunoblot analysis

Cells were washed twice with ice-cold PBS and scraped off the culture plate. After centrifugation at 6000 r.p.m. for 5 min and removal of the supernatant, cell pellets were re-suspended in lysis buffer (0.5% Triton X-100 in PBS, 0.5 mM PMSF and complete protease inhibitor mixture, Roche Applied Sciences, Mannheim, Germany) and incubated on ice for 30 min followed by a sonification with an amplitude of 8% for 10 s. Clear lysates were obtained after centrifugation for 5 min at 6000 r.p.m. Protein concentrations were determined using the BCA method (Molecular Probes). Equal amounts of protein were boiled for 5 min in 2 × SDS sample buffer and subjected to 10 or 12% SDS–PAGE electrophoresis at 80 mA before transferred to a polyvinylidenedifluoride membrane, which had to be activated in methanol before. Transfer lasts 1 h and 30 min at 350 mA and was followed by blocking in 4% skimmed milk in 0.05% Tween 20/Tris-buffered saline (TBS-T) for 30 min before incubation with primary antibody. The dilutions for primary antibodies are described in Supplementary Table 1. After incubating the primary antibody overnight at 4 °C under gently shaking, membranes were washed three times with TBS-T for 10 min each and incubated with the appropriate horseradish peroxidase-conjugated secondary antibody for 1 h (antibody dilution 1:10 000) followed by the same washing procedure. Mutant SigR1 aggregates were detected by using dot blot analyis. For this, lymphoblastoid cell lysates were directly brought on a nitrocellulose membrane and after applying a vacuum, membranes were processed following our established western blot protocol. Immunoreactive proteins were detected by enhanced chemiluminescence (Amersham Biosciences, Mannheim, Germany). Densitometric quantification of the band intensity was normalized to tubulin levels using Adobe Photoshop CS5.

### EGFR endocytosis, starvation and degradation assay

For EGFR endocytosis and degradation analysis, A431 cells were starved in DMEM without serum for 4 h. After starvation, cells were treated with EGF (100 mg) to stimulate EGFR endocytosis. Cells were then collected at various time points (as indicated in Figures 3l and m) and lysed in RIPA buffer (50 mM Tris–HClpH 7.5, 1% Triton X-100, 150 mM NaCl, 1 mM EDTA and 0.1% Na deoxycholate) containing protease inhibitor. Protein extracts were resolved by SDS–PAGE and immunoblotted using anti-EGFR antibody.

### EGFR surface biotinylation assay

Surface biotinylation assays were performed as previously described^14^ with minor modifications. Briefly, transfected A431 cells were starved in DMEM without serum for 4 h. After starvation, cells were treated with EGF to stimulate EGFR endocytosis and then preceded for surface biotinylation assay according to the manufacturer's protocol.

### Mito-red staining

MCF-7 cells were grown on coverslips and transfected with wtSigR1 or mSigR1. After 48 h cells were incubated with the mito-red dye for 10 min at 37 °C and processed according to the manufacturer's protocol for adherent cells. For quantification of enlarged mitochondria mito-red stained cells were visualized with the LSM 700 confocal microscope using the 40 × lens. Enlarged mitochondria (showing accumulation of mito-red staining and larger in size than normal mitochondria) from 10 random fields and at least capturing 8–10 cells per field of view were counted manually.

### CYTO-ID Autophagy detection

MCF-7 cells were grown on coverslips and transfected with wtSigR1 or mSigR1. After 48h cells were incubated with the CYTO-ID dye (Enzo, Lausen, Switzerland) for 30 min at 37 °C and processed according to the manufacturer's protocol for adherent cells. Lymphoblastoid cells derived from E102Q-SigR1 fALS patients and healthy controls were processed following the manufacturer's instructions for suspension cells. Both were analyzed by confocal microscopy.

### Enzyme activity assays and cell viability

NSC-34 cells were transfected with pcDNA, wtSigR1 or mSigR1 as described previously. After 48h incubation, cells were washed twice with ice-cold PBS and scraped off the culture plate using a cell scraper. Cell pellets were lysed in homogenization buffer for 15 min (10 mM Tris-HCl (ph 7.4), 1 mM EDTA, 5 mM DTT, 5 mM ATP, 20% glycerol and complete protease inhibitor mixture, Roche Applied Sciences) and processed for the proteasome activity assay (Jana). Proteasome activity was measured by using the proteasome substrate Suc-Leu-Leu-Val-Tyr-AMC. Lymphoblasts obtained from patients or healthy control individuals were processed for the caspase-3 activity assay following the same protocol using the caspase-3 substrate N-Acetyl-Asp-Glu-Val-Asp-7 amido-4-methylcoumarin.

Cell viability (mitochondrial activity) was determined by using Alamar Blue. Therefore, NSC34 cells were grown in 24 well plates and transfected according to the protocol described before. Alamar Blue was added to the medium in a 1:10 dilution and absorbance was measured after 2 h at 570 nm.

### Cellular fractionation

The subcellular distributions of distinct proteins were determined by using a subcellular fractionation kit following the manufacturer's protocol (Thermo Scientific/Life Technologies) and analyzed by immunoblotting. The subcellular fractionation was performed with patients' and control lymphoblasts as well as with NSC34 cells transfected according to the described procedure.

### Statistical analysis

We used the unpaired Student’s t-test for comparison between two sample groups. Values were expressed as mean±S.D. from three independent experiments. For Ca^2+^ measurements results were expressed as means±S.E.M. of ~30 cells. For the FRAP and co-localization experiments, we used the Mann–Whitney *U*-test. For both statistical tests, differences between the compared experimental conditions were regarded as significant when *P<0.05,**P<0.005.
