# Supplementary material for: The ALS-linked E102Q mutation in Sigma receptor-1 leads to ER stress-mediated defects in protein homeostasis and dysregulation of RNA-binding proteins
Source: Cell Death Differ. 2017 Jun 16;24(10):1655–71. doi: 10.1038/cdd.2017.88 (PMC5596426; doi:10.1038/cdd.2017.88)
Supplement: Supplementary Table [file cdd201788x1.docx]

**Supplementary table 1**

| **Antibodies** | **Source** | **Catalog Nr.** | **Dilution ( WB)** | **Dilution ( IHC/IF)** | **Species** |
| --- | --- | --- | --- | --- | --- |
| anti-KDEL | SDIX | 3154.00.02 | - | 1:250 | Rabbit |
| anti-calreticulin | Cell Signaling | #12238 | - | 1:250 | Rabbit |
| anti-emerin | Novocastra | Emerin-CE |  | 1:250 | Mouse |
| anti-β-Cop | Thermo Scientific | PA1-061 | - | 1:250 | Rabbit |
| anti-GM130 | BD Biosciences | 610822 | - | 1:250 | Mouse |
| anti-TDP43 | Abnova | H00023435-M01 | 1:1000 | 1:250 | Mouse |
| anti-Matrin 3 | Bethyl | A300-591A | 1:1000 | 1:250 | Rabbit |
| anti-Fus | Novus | NB100-2599 | 1:1000 | 1:250 | Rabbit |
| anti-Tia 1 | BD Biosciences | 610352 | - | 1:250 | Mouse |
| anti-p70 S6 kinase α | Santa Cruz | Sc-8418 | - | 1:250 | Mouse |
| anti- Lamin A | Vector | VP-L550 | - | 1:250 | Mouse |
| polyclonal anti-ubiquitin | Dako | Z0458 | 1:1000 | - | Rabbit |
| Anti-Erlin 2 | Sigma Aldrich | HPA002025 | 1:1000 | - | Rabbit |
| anti-RFP | MBL |  | 1:1000 | - | Rabbit |
| anti-Lamp 1 | Sigma Aldrich | L1418 | 1:1000 | - | Rabbit |
| anti-LC3 | Sigma Aldrich | L7543 | 1:2000 | - | Rabbit |
| anti-p62 | MBL | PM045 | 1:2000 | 1:500 | Rabbit |
| anti- EGFR | Cell Signaling | #64952 | 1:2000 | - | Rabbit |
| anti-Sigma receptor-1 | Santa Cruz | Sc-137075 | 1:500 | 1:250 | Mouse |
| anti-Sigma receptor-1 | Proteintech | 15168-I-AP | 1:500 | 1:250 | Rabbit |
| anti-GRP78 | BD Biosciences | 610978 | 1:2000 | 1:500 | Mouse |
| anti-peIF2α | Cell Signaling | #3597 | 1:500 | - | Rabbit |
| anti-EIF2α | Santa Cruz | Sc-133132 | 1:1000 | - | Mouse |
| anti- Caveolin- 1 | Santa Cruz | Sc-894 | 1:500 | - | Rabbit |
| anti-Hsp70 | Millipore | MAB3516 | 1:1000 | 1:500 | Mouse |
| anti-PDI | Cell Signaling | #3501 | 1:500 | - | Rabbit |
| anti- Rab5 | Sigma Aldrich | R4654 | 1:1000 | 1:500 | Rabbit |
| anti- Rab7 | Sigma Aldrich | R8779 | 1:1000 | 1:500 | Rabbit |
| anti- EEA1 | BD Biosciences | 610456 | 1:500 | - | Mouse |
| anti-α tubulin | Sigma Aldrich | T5168 | 1:10000 | - | Mouse |
| anti-GADD 153 | Santa Cruz | Sc-793 | 1:1000 | - | Rabbit |
| anti-Cytochrome C | BD Biosciences | 556432 | - | 1:250 | Mouse |
| anti-COX4 | Novus | NB110-39115 | - | 1:250 | Rabbit |
| anti-Tim23 | BD Bioscience | 611223 | - | 1:250 | Mouse |
| anti- Stim 1 | Sigma Aldrich | HPA011088 | 1:1000 | - | Rabbit |
| anti- VAPB | Home made |  | 1:1000 | - | Rabbit |
