# Supplementary material for: The ALS-linked E102Q mutation in Sigma receptor-1 leads to ER stress-mediated defects in protein homeostasis and dysregulation of RNA-binding proteins
Source: Cell Death Differ. 2017 Jun 16;24(10):1655–71. doi: 10.1038/cdd.2017.88 (PMC5596426; doi:10.1038/cdd.2017.88)
Supplement: Supplementary Figure and Table Legends [file cdd201788x3.docx]

**Suppl. Figure legends**

**Suppl. Figure 1: mSigR1 accumulates in the ER and induces ER stress-mediated cellular toxicity**

**(A)** SigR1 immunoblot to determine endogenous SigR1 protein expression by the cell lines used in this study in the untransfected state.

**(B-C)** MCF-7 cells **(B)** or NSC-34 cells **(C)** were transfected with either pcDNA, wtSigR1 or mSigR1 and processed for SigR1 immunofluorescence. Note the SigR1 aggregation and shrinkage of the cells expressing mSigR1 protein. Scale bar = 15 µm.

**(D)** Immunoblot analysis for established ER stress and autophagy markers in MCF-7 cells and **(F)** NSC34 cells over-expressing wtSigR1 or mSigR1 at increasing concentrations ranging from 0.25 to 1.5 µg.

**(E, G)** Quantification of the MCF-7 and NSC-34 cells´ immunoblots depicted in **(D, F).**

**(H)** Immunofluorescence staining with antibodies against SigR1 and Tim23 as a mitochondrial marker.

**(I)** NSC-34 cells were transfected with pcDNA, wtSigR1 and mSigR1 as described above and cell lysates were processed for chymotrypsin-like proteasomal activity or caspase-3 activity. Values are expressed as mean ± SD of three independent experiments. *p<0.05.

**Suppl. Figure 2: RT- PCR analysis of MCF-7 cells over-expressing wtSigR1 and mSigR1**

**(A-D)** RT-PCR analysis for UPR markers (ATF4, ATF6 and XBP1). 48 h post-transfection with either wtSigR1 or mSigR1 **(A)**, at different time intervals **(B)** and at variable concentrations of transfectant **(C**), in lymphoblastoid cells from three healthy controls and two E102Q-SigR1 fALS patients **(D)**. The asterisks (*) denote significant differences (**p*<0.05), while # denotes absence of significance.

**(E)** Lymphoblastoid cells from three healthy controls and two E102Q-SigR1 fALS patients were processed for chymotrypsin-like proteasomal activity or caspase-3 activity. Values are expressed as means ± SD of three independent experiments. *p<0.05.

**Suppl. Figure 3:**

**(A-D)** MCF-7 cells expressing wtSigR1 or mSigR1 were processed for EM. **(A)**  Expression of mSigR1 leads to the accumulation of vacuoles probably derived from the ER. **(B)** They often contain non-degraded cargo surrounded by double membranes (arrwoheads) characteristic for autophagic vacuoles (AV). **(C, D)** wtSigR1 expressing cells show normal appearing ER (white arrows) and nuclear envelope, whereas expression of mSigR1 leads to ER widening (white arrows), associated with mitochondrial degeneration (grey arrows) and protrusions of the nuclear envelope (black arrows)**.**

**Suppl. Figure 4:**

**(A)** Immunoblot analysis of MCF-7 cells transfected with either pcDNA, wtSigR1 or mSigR1 using the autophagy markers p62 and LC3.

**(B)** 48 h after transfection, A431 cells were starved for the indicated periods of time by replacing the normal medium with starvation medium (see methods for detail).

**(C)** Transiently transfected MCF-7 cells were additionally treated with Rapamycin (2 µM) or Bafilomycin A (2 µM) for 4 h to accelerate or inhibit autophagy and then processed for immunoblot analysis.

**(D)** MEF GFP-LC3 cells expressing pcDNA, wtSigR1 or mSigR1 were processed for immunoblot analysis using the autophagy markers p62 and LC3.

Corresponding densitometric analysis are shown below each panel of **(A, B, C, D).** Values are expressed as means ± SD from three independent experiments. The asterisks (*) denote significant differences (**p*<0.05), while # denotes absence of significant differences.

**(E)** A431 cells were transiently transfected with pcDNA, wtSigR1 or mSigR1. After 48 h, cells were processed for surface biotinylation assays. Immunoblot analysis using EGFR antibody revealed no significant alteration in EGFR internalization.

**(F)** Immunofluorescence showing no co-localization of SigR1 aggregates and LAMP1 (a specific marker for lysosomes) in E102Q-fALS patient and healthy control lymphoblastoid cells.

**(G)** Immunoblot analysis of matrin-3 in subcellular fractions obtained from MCF-7 cells expressing pcDNA, wtSigR1 or mSigR1. The corresponding densitometric analysis is shown below. The asterisks (*) denote significant differences (**p*<0.05),

**(H)** Primary fibroblasts from autophagy reporter GFP-LC3 transgenic mice (MEF GFP-LC3) were transiently transfected with mSigR1. Anti-TDP-43 immunofluorescence reveals extra-nuclear translocation of TDP-43 (white arrowheads in lower panel). Note the granular GFP-LC3 accumulation in mSigR1 over-expressing cells. Scale bar = 10 µm.

**(I-J)** Immunofluorescence staining showing no co-localization of SigR1 aggregates and S6-kinase (a specific marker for p-bodies) in E102Q-fALS patients´ lymphoblastoid cells **(I)** and in mSigR1-expressing MCF-7 cells **(J).** Scale bars =15 µm.

**Suppl. Figure 5: TDP-43 and matrin-3 in sALS spinal cord**

**(A-B)** Representative immunohistochemical pictures showing co-labelling of pTDP-43 (red) with matrin-3 (green) in lumbar α-motor neurons of control and sALS cases. Scale bar = 15 µm.

**(C-D)** NSC34 cells were treated with the autophagy inhibitor Bafilomycin A (2 µM), MG132 (2 µM), Thapsigargin (2 µM), Rapamycin (2 µM) and PRE-084 for 4 h. Subcellular fractions were processed for immunoblot analysis with matrin-3 **(C)** and TDP-43 **(D)** antibodies. Note the cytoplasmic translocation of matrin-3 especially after Bafilomycin treatment.

**(E)** Coomassie staining of the subcellular fractions as a loading control for the immunoblot analysis depicted in **(C-D)**.

**Supplementary Table 1:** List of all the primary antibodies (Suppl. Table 1) used in the manuscript including their source, dilutions and catalog number.

**Movie legends**

**Movie 1.** mSigR1 impairs vesicle transport from ER to Golgi. Note the slower recovery of the fluorescence signal within the bleached area in mSigR1-expressing cells as compared to control cells. Play back speed is approximately 15-fold of the real speed.
